# The relationship of adverse childhood experiences to a history of premature death of family members

**DOI:** 10.1186/1471-2458-9-106

**Published:** 2009-04-16

**Authors:** Robert F Anda, Maxia Dong, David W Brown, Vincent J Felitti, Wayne H Giles, Geraldine S Perry, Edwards J Valerie, Shanta R Dube

**Affiliations:** 1 ACE Study Group. National Center for Chronic Disease Prevention and Health Promotion, Centers for Disease Control and Prevention, Atlanta, Georgia, USA; 2 Department of Preventive Medicine, Southern California Permanente Medical Group (Kaiser Permanente), San Diego, California, USA

## Abstract

**Background:**

To assess the association between adverse childhood experiences (ACEs), including childhood abuse and neglect, and serious household dysfunction, and premature death of a family member. Because ACEs increase the risk for many of the leading causes of death in adults and tend to be familial and intergenerational, we hypothesized that persons who report having more ACEs would be more likely to have family members at risk of premature death.

**Methods:**

We used data from 17,337 adult health plan members who completed a survey about 10 types of ACEs and whether a family member died before age 65. The prevalence of family member premature death and its association with ACEs were assessed.

**Results:**

Family members of respondents who experienced any type of ACEs were more likely to have elevated prevalence for premature death relative to those of respondents without such experience (p < 0.01). The highest risk occurred among those who reported having been physically neglected and living with substance abusing or criminal family members during childhood. A powerful graded relationship between the number of ACEs and premature mortality in the family was observed for all age groups, and comparison between groups reporting 0 ACE and ≥ 4 ACEs yielded an OR of 1.8 (95%CI, 1.6–2.0).

**Conclusion:**

Adverse childhood experiences may be an indicator of a chaotic family environment that results in an increased risk of premature death among family members.

## Background

Increasing the life expectancy of Americans of all ages is the first goal of Healthy People 2010 set forth by the United States Department of Health and Human Services [1]. However, mortality from the leading causes of death (eg, heart disease, cirrhosis, unintentional injury, suicide, homicide, and HIV/AIDS) among adults continues to pose a major obstacle in achieving this goal [2-6]. Various external health risk factors, such as tobacco, diet and activity patterns, alcohol, sexual behavior, motor vehicle crashes, and use of illicit drugs contribute prominently to the leading causes of death [7].

Many of the risk factors for leading causes of death in adults are associated with traumatic childhood experiences. Growing up experiencing childhood abuse, neglect, and growing up with serious forms of family dysfunction substantially increases the risk of smoking, illicit drug abuse, alcoholism, suicide and perceived poor health (which predicts early death) in adults [8-14]. Thus, the leading causes of death in adults are strongly related to the experiences of childhood.

Structured family roles promote social control of family members' health behaviors which, in turn, affect subsequent mortality later in life [15]. In turn, dysfunctional families will expectedly lose their ability to modulate unhealthy risk behaviors [16]. For example, compared to married people or non-alcohol abusers, the divorced, separated, or alcohol abuser have shorter life expectancy and higher morality rates, particularly from deaths that have large psychological and behavioral components, such as suicide, accidents, lung cancer or cirrhosis [17-21]. Individuals who live in households where other members use illicit drugs have an increased risk of violent death [22,23]; and women who live in a household where domestic violence and substance abuse are present are at particularly high risk of homicide at the hands of a spouse, an intimate acquaintance, or a close relative [23].

Taken together, this information leads us to expect higher rates of premature mortality (before age 65 years) among the family members of persons who grew up with adverse childhood experiences. Yet, the complex pathways by which a dysfunctional family environment affects early mortality are not clearly defined. Further, no conclusion about the impact and potential relevance of an individual's traumatic childhood experiences to the risk of premature mortality among family members can be drawn [24]. However, given that there is evidence that violence and serious household dysfunction tends to be intergenerational [25-27] and that multiple members of a household would be adversely affected by them, we hypothesized that individual's reports of adverse childhood experiences would be an indicator of health risks in the extended family that potentially bridges generations.

To assess this hypothesis, we analyzed data from a large sample of health maintenance organization members who have provided information about childhood emotional, physical, or sexual abuse; emotional and physical neglect; growing up with domestic violence, and household members who were substance abusers, mentally ill, or criminals, and their reports of premature mortality among their family members.

## Methods

### Study participants and data collection

The Adverse Childhood Experiences (ACEs) Study is a retrospective cohort study conducted in San Diego, California at Kaiser Permanente's Health Appraisal Clinic. Annually at this clinic, more than 50,000 members undergo a standardized medical examination. In any 4-year period, some 80% of adult members complete the examination that is primarily for health assessment rather than symptom- or illness-based care. Thus, there is no reason to believe that selection bias is a significant factor in the Study [28]. Between 1995 and 1997, the ACE Study was carried out in two consecutive waves among 26,824 adult members. The response rates were 70% and 65% for Wave I and II respectively, and the overall response rate was 68% (n = 18 175). After excluding duplicate respondents who coincidentally underwent examinations for both waves (n = 754) and those missing information about race and education (n = 84), the final sample included 9,367 (54%) women and 7,970 (46%) men.

The ACE questionnaire, which was mailed to members two weeks after their medical examination, contained detailed questions about childhood abuse, neglect, and household dysfunction, as well as information about health-related behaviors and premature death of a family member. Questions about childhood experiences were framed as "While you were growing up during your first 18 years of life".

### Definitions

All ACE questions (Table 1) pertained to the first 18 years of life. Questions from the Conflict Tactics Scale [29] were used to define emotional and physical abuse and domestic violence. Questions on emotional and physical neglect were contained only in Wave II. They were adapted from the Childhood Trauma Questionnaire [30], and scored on a Likert scale. Childhood sexual abuse was assessed using four questions adapted from Wyatt [31], and was determined by a "yes" response to one or more of the questions.

**Table 1:** Definition and prevalence of adverse childhood experience and ACE score

Category of adverse childhood experiences (Total N = 17 337)	Prevalence (%)
Abuse	
Emotional abuse	10.6
*Did a parent or other adult in the household ever, sometimes, often or very often *(1) swear at you, insult you, or put you down? (2) act in a way that made you afraid that you might be physically hurt?	
Physical abuse	28.3
*Did a parent or other adult in the household often or very often *(1) push, grab, slap, or throw something at you? (2) hit you so hard that you had marks or were injured?	
Sexual abuse	20.7
*Did an adult or person ≥ 5 years older ever *(1) touch or fondle you or (2) have you touch their body in a sexual way? (3) attempt or (4) actually have oral, anal, or vaginal intercourse with you?	
Household dysfunction	
Domestic violence (*response options: never, once or twice, sometimes, often, very often*)	12.7
*Was your mother (or stepmother) *(1) pushed, grabbed, slapped, or had something thrown at her? (2) kicked, bitten, hit with a fist, or hit with something hard? (3) repeatedly hit over at least a few minutes? (4) threatened with or hurt by a knife or gun?	
Parental separation or divorce	23.3
Parents ever separated or divorced?	
Mental illness in household	17.3
*Lived with a household member who *was depressed or mentally ill?	
Household substance abuse	26.9
*Lived with anyone who *(1) was a problem drinker/alcoholic? (2) used street drugs?	
Criminal household member	4.7
Did a household member go to prison?	
Neglect*	
Emotional neglect	14.8
(1) There was someone in my family who helped me feel important or special. (2) I felt loved. (3) People in my family looked out for each other. (4) People in my family felt close to each other. (5) My family was a source of strength and support.	
Physical neglect	9.9
(1) I didn't have enough to eat. (2) I knew there was someone there to take care of and protect me. (3) My parents were too drunk/high to take care of me. (4) I had to wear dirty clothes. (5) There was someone to take me to the doctor if I needed it.	
ACE score (Number of Adverse Childhood Experiences)	
0	36.4
1	26.2
2	15.8
3	9.5
≥ 4	12.1

* The data are available in Wave II only (N = 8629). Some items in both neglect categories were reverse scored to reflect the framing of the question.

Unlike previous ACE Study analyses, suicide attempts among family members was not included as an adverse childhood experience in the category of mental illness of household members. We made this change for this analysis to eliminate the upward bias to a relationship between ACEs and a family member's premature death due to suicide.

To assess the cumulative effect of multiple ACEs, we summed the total number of categories of ACEs reported to generate the ACE score (range: 0–8). The ACE score did not count childhood emotional and physical neglect because they were collected only in the second survey wave.

Premature death in the family was defined as a "yes" response to the question: "Have members of your family died before the age of 65?" This question was a part of the standardized medical evaluation and was not framed as an adverse childhood experience.

We further examined other family risk factors collected and assessed their relationship with premature death of the family member. They included information about whether their parents smoked, whether a family member had been murdered, attempted suicide, or had heart disease before age of 60 ("Has a parent, brother, or sister developed coronary (heart) disease before age 60?").

### Statistical analyses

We examined both the relationship of each individual ACE and the ACE score to the risk of premature death of family member. All analyses were performed using SAS [32]. Adjusted prevalence of premature death in the family was calculated using general linear models and the risk of premature death for all ten ACE categories and levels of the ACE score were determined from adjusted odds ratios (ORs) estimated using multivariate logistic regression. In the logistic models, the ACE score (1, 2, 3, ≥ 4) was entered as a set of dummy variables and all models accommodate adjustments for four demographic covariates: age at survey, sex, race, and education. Education, measured as highest grade completed, was categorized into four levels: not completed high school, high school diploma, some college, or college graduate. To assess possible differences in association by age, participants were divided into four age groups: 19–34, 35–49, 50–64, and 65 years and older; and adjustment by single years of age within each age category was included in the regression model. Using SAS diagnostics, we found no evidence of collinearity between the ACEs and demographic factors.

Because of the reported substantial impact of socioeconomic factors on overall mortality [33], three additional covariates were considered: home ownership during childhood, and paternal and maternal educational attainment, both of which were included in wave I and II separately. These three socioeconomic variables, however, were subsequently excluded from the final analyses because their inclusion in the regression models added little to the proportion of variance explained and did not significantly altered either the relationship of each ACE category or the number of ACEs to family member premature death.

Persons with missing information about an ACE were considered not to have had that experience. Theoretically, this would slightly attenuate the relationship between ACEs and risk of premature death in the family because some persons who had potentially been exposed to an experience would always be classified as unexposed. To assess the potential effect of this assumption, we repeated the analyses after excluding respondents with missing information on any of the ACEs and found no differences in the results.

## Results

The mean age in years for participants was 56 (SD, 15.2). Seventy-five percent of participants were white and 39% were college graduates. Only 7% had not graduated from high school. Almost one third of the respondents (28.3%) reported childhood physical abuse; 20.7% reported having been sexually abused during childhood; 26.9% reported having a family member abuse drugs or alcohol; and 12.7% witnessed domestic violence as a child (Table 1). Overall, nearly two third (63.6%) of respondents had been exposed to at least one type of ACE; and 12.1% exposed to four or more types of ACEs (Table 1).

### Prevalence of premature death in the family

Overall, 47% of respondents reported having a family member who died before age 65; female respondents reported slightly higher rates (50%) than males (43.5%). Blacks reported the highest prevalence (59.4%) compared with respondents from other ethnic groups. After controlling for other demographic covariates, the prevalence of premature death in the family increased with respondent's age at survey, but decreased with respondent's education (Table 2).

**Table 2:** Prevalence of reporting a family member having died before 65 years by demographic characteristics

Demographic variables	Number of subjects	Crude prevalence (%)	Adjusted prevalence (%)*	SE	P value
Sex					
Female	9367	49.7	50.0	0.5	Referent
Male	7970	43.9	43.5	0.6	< 0.0001
Race					
White	12 964	46.9	46.1	0.4	Referent
Black	789	57.5	59.2	1.8	< 0.0001
Hispanic	1942	43.7	45.9	1.2	0.86
Asian	1244	45.7	48.5	1.4	0.095
Am Indian	63	49.2	49.2	6.2	0.6238
Other	335	49.3	52.4	2.7	0.024
Education					
No high school diploma	1251	52.0	49.7	1.4	Referent
HS graduate	3044	48.7	46.7	0.9	0.08
Some college	6220	48.3	48.5	0.6	0.45
College	6822	44.2	45.3	0.6	0.0056
Age group					
19–34	1721	36.5	35.2	1.2	Referent
35–49	4494	42.8	42.6	0.7	< 0.0001
50–64	5534	47.9	48.0	0.7	< 0.0001
65 and older	5588	52.7	53.2	0.7	< 0.0001
Father's education†					
No high school diploma	3611	51.2	49.3	0.9	Referent
HS graduate	1918	45.9	46.6	1.1	0.0611
Some college	1394	45.3	46.6	1.3	0.0972
College	1306	39.5	42.3	1.4	< .0001
Mother's education†					
No high school diploma	3542	50.1	47.8	0.9	Referent
HS graduate	2549	48.3	49.4	1.0	0.2239
Some college	1388	42.6	44.4	1.4	0.0440
College	867	39.3	42.3	1.7	0.0077
Home ownership‡					
Yes	6004	45.2	45.7	0.6	Referent
No	2497	51.9	50.7	1.0	< .0001
Total	17 337	47.0			

*Adjusted for education, sex, race and age at the survey.

†Available only in wave II data only (total n = 8629; father's education, n = 8229; mother's education, n = 8346)

‡ Defined by question: "For most of your childhood, did your family own their home?" This information available only in wave I (total n = 8501).

Higher family socioeconomic status, reflected by a higher education level of the mother or father, and owning a house during childhood, was related to lower prevalence of the family premature death (Table 2).

### Association between ACEs and premature death in the family

The adjusted prevalence of premature death in the family for persons who reported each category of ACE was significantly higher than for those without such exposure (Table 3). The adjusted ORs for premature death in the family for each individual ACE ranged from 1.1 for emotional abuse and parental marital discord (95% CI, 1.1–1.2) to 1.6 for growing up with a criminal in the home (95% CI, 1.4–1.9) (Table 3). We found a strong graded relationship between the number of ACEs (ACE score) and the adjusted prevalence and risk (ORs) of premature death in family (Table 3).

**Table 3:** Crude and adjusted prevalence of premature death in the family for each adverse childhood experience and ACE score

Category of ACE	N	Crude prevalence (%)	Adjusted prevalence (± SE) (%)*	Adjusted OR	95% CI
Abuse					
Emotional abuse					
No	15 508	46.9	46.7 (0.4)	1.0	Referent
Yes	1829	48.5	49.8 (1.1)	1.2	1.1–1.3
Physical abuse					
No	12 425	46.0	45.5 (0.4)	1.0	Referent
Yes	4912	49.6	50.8 (0.7)	1.2	1.2–1.3
Sexual abuse					
No	13 751	45.8	45.7 (0.4)	1.0	Referent
Yes	3586	51.9	52.0 (0.8)	1.3	1.2–1.4
Household dysfunction					
Domestic violence					
No	15 136	46.2	46.1 (0.4)	1.0	Referent
Yes	2201	52.6	53.2 (1.1)	1.3	1.2–1.5
Parental separation or divorce					
No	13 306	46.5	46.2 (0.4)	1.0	Referent
Yes	4031	48.8	49.6 (0.8)	1.1	1.1–1.2
Mental illness in household					
No	13 978	46.4	46.1 (0.4)	1.0	Referent
Yes	3359	50.2	51.6 (0.9)	1.3	1.2–1.4
Household substance abuse					
No	12 425	44.9	44.2 (0.4)	1.0	Referent
Yes	4912	52.7	54.8 (0.7)	1.5	1.4–1.7
Criminal household member					
No	16 528	46.6	46.5 (0.4)	1.0	Referent
Yes	809	56.7	58.2 (1.8)	1.6	1.4–1.9
Neglect**					
Emotional neglect					
No	7355	46.5	46.1 (0.6)	1.0	Referent
Yes	1274	51.0	51.3 (1.4)	1.2	1.1–1.4
Physical neglect					
No	7774	46.1	46.1 (0.6)	1.0	Referent
Yes	855	56.8	56.9 (1.7)	1.5	1.3–1.8
ACE score (Number of ACEs)					
0	6078	43.9	42.4 (0.6)	1.0	Referent
1	4472	46.7	46.6 (0.7)	1.2	1.1–1.3
2	2706	47.2	48.2 (0.9)	1.3	1.2–1.4
3	1681	49.8	51.3 (1.2)	1.4	1.3–1.6
≥ 4	2400	54.6	56.8 (1.1)	1.8	1.6–2.0

* Adjusted for education, age, sex and race.

** Wave 2 data only (N = 8629).

**Figure 1:**
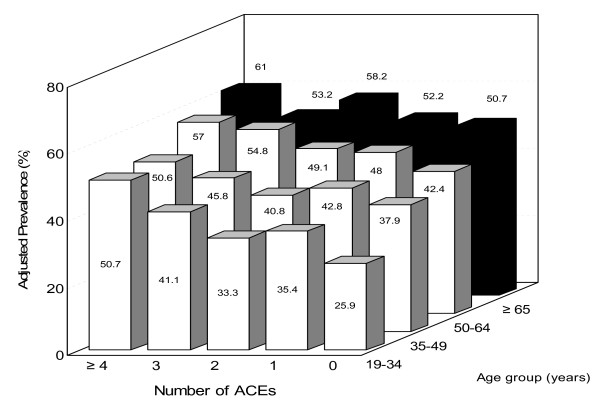
**Adjusted prevalence of premature death of family member among different age groups by number of ACEs**.

The graded relationship between the ACE score and the adjusted prevalence of premature death in the family was consistently strong for all 4 age groups (Figure 1). The variation of prevalence of premature death in the family between respondents with an ACE score of 4 or more and those with ACE score of 0 was highest in the youngest age group (15–34 years); and these differences for persons with ACE scores of 0 or ≥ 4 gradually diminished with increasing age. In logistic regression models, the strongest relationship was again observed among respondents who were aged 19–34 years, for whom comparison between reporting 0 ACE and reporting ≥ 4 ACEs yielded an OR of 2.9 (95%CI, 2.1–4.0) whereas the ORs were 1.7 (95%CI, 1.4–2.0), 1.8 (1.5–2.2), and 1.5 (1.1–2.0) for persons in the 35–49, 50–64 and ≥ 65 year age groups, respectively.

To examine the significance of the graded relationship between the ACE score and the risk of family member premature death, the ACE score was entered as an ordinal variable into logistic regression models, with adjustment for covariates. The ordinal OR was 1.13 suggesting that for every increase in the ACE score the likelihood of reporting premature death of family member increased by 13% (*P *< .001). The test for trend between ACE score and the risk of premature death was significant in all four age groups (*P *< .05; data not shown).

In separate analyses, we found the risk for family member premature death was significantly elevated for other related risk factors among family members. The risk (ORs) of reporting a family member having died before age 65 was 1.9 (95%CI, 1.3–3.0) among respondents who reported a family member who had been murdered, and 1.6 (1.4–1.8) for attempted suicide, 2.9 (2.7–3.2) for having had a heart attack before age 60, and 1.2 (1.2–1.3) for parental smoking, relative to those who did not report such an event among their family members. A graded relationship between ACE score and these risk factors among family members was observed, consistent with the findings for the relationship between ACEs and premature death in the family (data not shown).

## Discussion

We found a strong graded relationship between the ACE score and the risk of premature death of a family member. This relationship was consistent for each of four successive birth cohorts suggesting that the finding is not related to secular trends or to the amount of time a respondent had to "observe" premature death in the family. In addition, each of the individual categories of adverse childhood experiences was also associated with an increased risk for premature death of a family member. These findings are consistent with related studies that linked risks for premature death, such as heart diseases, substance abuse, and attempted suicide among individuals who reported these ACEs [9-14].

The mechanisms for association observed between ACEs and premature death in the family merit serious consideration. First, the impact of ACEs on subsequent family member premature mortality may take place through the combined effects of social and biological risk occurring at different life stages, such as neurological development during childhood, and health behaviors inculcated during adolescence and adulthood [34,35]. Thus, the entire family (i.e., members of all ages) would be affected. Moreover, family relationships may provide social control of health behaviors indirectly by affecting the internalization of norms for healthy behavior, and sanctions for deviating from behavior conducive to health [15-17,36,37]. Multiple ACEs indicate a disordered, stressful social environment that can decrease, eliminate, or reverse these favorable impacts of social control. Family members living in such environments are more likely to have been exposed to persons engaging in undesirable health practices such as smoking, illicit substance use, excessive drinking, or unsafe sexual practices, which may have resulted from responses to traumatic stress (ACEs) [15,37].

Second, ACEs are likely to contribute to an intergenerational cycle of risks [38,39]. Evidence suggests that certain ACE related conditions may be "transmitted" intergenerationally. For example, early maternal and paternal age has been found among the second generation of teenage mothers [40]. Several studies also reported association of mental illness between generations [41-43]. Thus, persons with high ACE scores may be more likely to have parents, or even children with high ACE scores. If such is the case, the relationship of ACEs to many of the leading causes of death may account for the graded relationship of the ACE score to the premature death of a family member. In addition, other changes in family status that are associated with ACEs, such as a suicide or homicide of a family member or changes in residence [14,45] may be precipitants of behaviors or physiologic changes that may result in early death.

Finally, some ACE-related risk factors appear to be interrelated in increasing the early death of the family member. For example, depressed people are more likely to commit suicide, illicit drug users were more likely to die a violent death, and alcoholics were more likely to be involved in fatal accidents. This was further evidenced by our findings that parental smoking, family member suffering a heart attack before age 60, being murdered, or having attempted suicide were strongly associated both with the number of ACEs and with the increased risk of family member premature death.

Although economic well-being is considered to be a basic explanation for mortality overall [33] the adjustment for respondents' present or childhood socioeconomic position had little influence on the findings in either the current study on family member premature death or the previous investigation of other health risks for the leading causes of death [9-14], rendering a simple explanation in terms of socioeconomic confounding less likely.

Several aspects of our study strengthen our findings. First, participants reported their family history during a routine clinical examination that preceded the survey about ACEs, reducing the possibility of bias toward attributing premature death of family member to ACEs. Second, population-based studies found levels of exposure to certain ACEs nearly identical to ours [46,47], suggesting our findings are likely to be applicable in other study settings. Third, a wide range of interrelated ACEs was studied, enabling us to assess the relationship of each ACE to the risk of premature death.

The prevalence of childhood exposures we reported is nearly identical to those reported in population-based surveys in North America. We found that 16 percent of the men and 25% of the women met the case definition for childhood sexual abuse, similar to findings by Finkelhor et al. that 16% of men and 27%of women had been sexually abused [48]. As for physical abuse, 28% of the men from our study reported experiencing this as boys, which closely parallels the percentage found (31%) in a recent population-based study of Ontario men that used questions from the same scales [49]. The similarity of the estimates from the ACE study to those of population-based studies suggests that our findings are likely to be applicable in other settings.

The graded relationship of the ACE score to premature death of a family member is particularly strong for younger respondents and diminishes with increasing age of the respondents (Figure 1). The decreasing strength of this relationship is likely due to the fact that the prevalence of premature death in the family increased with increasing age of the respondent (Table 1), which was expected because older respondents have had more time to experience the premature death of a family member and because they have lived through periods of time wherein life expectancy was lower relative to the periods of life experience for younger respondents.

Data from the National Mortality Followback Survey indicate that reporting of the age of death of a family member is quite accurate. In fact, the agreement between age at death based on death certificate information and age at death based upon reports from next of kin show 92.7% agreement for ages within 1 year of actual age at death [50].

Several potential limitations need to be considered when interpreting our results. First, our estimates of prevalence of family member premature death have limitations since certain information is unavailable regarding the family members, such as sex, age at death, employment, education, and differences in life circumstances. Second, since our question about family member premature death did not gather information regarding how many premature deaths occurred in a family, and our analysis treated any number of deaths as one single event, we could not estimate actual rates of premature death in the family; thus the prevalences we report almost certainly represent underestimates of the actual risk for premature death. Third, our findings were based on a retrospective survey which made it difficult to distinguish the causal impact of ACEs on premature mortality. In theory, prospective studies related to child maltreatment would avoid these potential biases. In practice, given the social and legal implications, this type of studies are nonetheless difficult to conduct [51].

It is possible that ACEs pose increased risk not only for respondents' health but also the premature death of their family members. This possibility has significant policy implications and suggests that ACEs may play an important role as a marker for hidden risk factors existing within the family that place individuals at risk of early death. These findings may also improve our understanding of the etiology of certain diseases and thus help in the formation of future preventive initiatives. The problem of ACEs in our society is a complex one and to adequately address this problem will require a multifaceted solution that may ultimately lead to decompression of premature mortality. Advocacy for the needs of one of our most vulnerable population, children, and subsequent generations will, no doubt, become increasingly important.

## Conclusion

The ACE score has a graded relationship to the prevalence of premature death of a family member. Thus, individual reports of adverse childhood experiences may be an indicator of familial and intergenerational risk of ACEs that increase risk of premature death among family members.

## Competing interests

The authors declare that they have no competing interests.

## Authors' contributions

MD planned the study, analyzed the data, and wrote the manuscript. RFA and VJF were the co-investigators who designed the Adverse Childhood Experiences Study, supervised the data collection, and assisted in writing of the manuscript. WHG, DWB, GSP, VJE and SRD assisted in writing of the manuscript.

## About the Authors

Maxia Dong, Robert F. Anda, Wayne H. Giles, David W. Brown, Geraldine S. Perry, Valerie J. Edwards, Shanta R. Dube are with the Division of Adult and Community Health, Centers for Disease Control and Prevention, Atlanta, Ga. Vincent Felitti is with the Department of Preventive Medicine, Kaiser Permanente, San Diego, California, USA. All authors read and approved the final manuscript.

## Pre-publication history

The pre-publication history for this paper can be accessed here:


